# Report of the Meeting of the Pennsylvania State Dental Society

**Published:** 1883-11

**Authors:** 


### REPORT OF THE MEETING OF THE PENNSYLVANIA STATE DENTAL SOCIETY, HELD AT CRESSON, JULY 31, AND AUGUST 1 AND 2, 1883.

(Concluded from the October Number.)

SECOND DAY, AUGUST 1ST. MORNING SESSION.

Dr. Darby, Chairman of the committee to prepare resolutions on death of Dr. Marshall H. Webb, presented a report, which was accepted, and ordered to be entered upon the minutes.

Dr. Magill presented the report of the committe on "Enforcement of Dental Law." They had prosecuted one case to a final issue.

The verdict was against the defendant, who afterwards attended a dental college and graduated.

The committee desired to have the sense of the meeting as to whether the term of pupilage should be counted as "time of practice." A case had occurred where the attention of the committee had been called to a dentist who was violating the law. The committee notified him, and he replied that the law did not apply to him, as he was still a student. Several years passed, and he then claimed that he had been in "practice" all that time and was exempted. The law does not say how long a man may be a student. Now, can a dentist continue to practice year after year and escape the penalty of the law by claiming to be a student as long as it suits his purpose, and then turn round and claim all that time as having been spent in practice? The committee had found this a knotty question, and desired light upon it.

Dr. Jack moved that the period of pupilage should *not* be considered as time spent in practice. After a discussion, the motion was passed unanimously.

Dr. Jack, after explaining that the State Examining Board was appointed to meet the case of those who were in practice before the dental law was passed, and that their certificate was not intended in any sense to take the place of a regular diploma, and that complaint had been made that their certificate was too easily obtained, moved that a committee be appointed by the President to confer with the Examining Board, to devise if need be, rules and regulations to make the certificate of qualification more difficult of attainment. Passed, and committee appointed.

Dr. Jack, after reading the clause in the constitution referring to patents, (Art. 7, Sec. 2) said he thought the time had come for the profession to take a firm stand against all secret preparations. He did not desire to deny to any one the just reward of their labor; no one could object to a dentist manufacturing and selling a preparation or a filling material,or a remedy he may have invented; but he thought we should take the same stand the medical profession has, and insist that the formula and the process shall first be published. These secret preparations were a hindrance to our professional progress, and in the end a serious injury. A dentist may, after a great deal of research and experiment, devise an amalgam far better than any that has preceded it. If the formula and process were published, another knowing how it was produced may suggest a little change and make it still better. When it is kept a close secret it compels all investigators in that field to go over the same ground, and to try the same fruitless experiments before any advance can be made. He considered them more objectionable than patents; the patent simply reserved the right of control over the thing patented, but prevented no one from taking up the idea and improving it, but keeping the idea secret does.

He then offered resolutions disqualifying any one from becoming or remaining a member of the society who had a pecuniary interest in any secret stopping, or therapeutic agent, and denying representation to any college, if any teacher connected with it had such an interest in a secret preparation.

Dr. Guilford did not think it right, if one teacher in a college violated the rules of the society to punish all the others for his fault; he was fully in accord with the first part of the resolutions, but thought the latter part unjust.

Dr. Magill said that a law could only be enforced so far as it met public sentiment. He thought the constitution went as far as the sentiment of the society called for. A man had a right, a personal right to the result of his own labor, that should not be interfered with.

After a long discussion the resolutions were passed.

Dr. Guilford read a paper upon "Properties of Gold Foil." He spoke at some length of the properties of gold, dwelling especially upon "cohesion," and the difference between "cohesive" and "plain" gold. He took the ground that naturally, gold foil was cohesive, and supposed that it was passed through some process to destroy this property when plain or non-cohesive gold was desired. Assisted by a chemical expert, he had spent some time endeavoring to discover what the physical difference between the two forms of gold was, but without any satisfactory result. With the most delicate chemical tests he was not able to find even a trace of any of the various agents said to be used in preparing non-cohesive gold. He suggested that the cohesion of gold might be caused by the inter-locking of the crystals on its surface, and that the increased cohesion caused by annealing might be due to the heat, lifting up and rendering these crystals more prominent. While gold could be crystalized, he had never seen any sign of crystalization on the foil, and had never heard of any one who had, yet he could conceive of the crystals being so minute, or so situated, that the best microscope would not define them. He referred to a paper published by Dr. Black a few years ago, in which were detailed a series of experiments that seemed to show that gold foil was liable to have a thin film of various gases on the surface, which destroyed to a greater or less extent its cohesion. Dr. Black had found that, except sulphur and phosphorus, those most commonly met with could be driven off by heat. He also found that hydrogen gas had a strong affinity for gold, and that gold coated with it was protected from other and more injurious gases. He therefore suggested keeping a little ammonia in the gold drawer. Then the gold would be covered with a film of hydrogen that would protect it from contact of other gases, and when annealed, the hydrogen being readily expelled by heat, it left the gold in good working condition. Dr. Guilford thought the experiments of Dr. Black offered the best explanation of the difference noticed in the working properties of gold foil. He briefly noticed the peculiarities of full, semi, and non-cohesive gold foil, and hoped that future investigations might enable us to find on what the cohesiveness of gold really depends.

---

SECOND DAY—AUGUST 1st—AFTERNOON SESSION.

After receiving the report of the State Examining Board, and of several committees, the society adjourned to attend the clinics.

CLINICS.

Dr. W. Storer How illustrated his method of attaching artificial crowns.

Dr. W. G. A. Bonwill exhibited his dental engine and mechanical mallet.

Dr. C. H. Land, of Detroit, Mich., exhibited a new form of air chamber. He contends that the air chamber should cover at least four-fifths of the entire lingual surface of the palatine arch, and certain proportions of the alveolar. He recommends trimming the plaster cast on the outer portion of the alveolar ridge, especially any projecting points, so as to make it a comparatively level surface, his idea and design being to let the pressure of the plates bear directly on the outer portion of the ridge. His air chamber is of the usual shape, but made much thinner where it bears upon the center of the palate. When the plate is placed in the mouth it rests entirely on those portions of the ridge cut from the cast. This undue pressure is expected to cause absorption, so that in a short time a perfect fit is obtained. In the mean time, until the plate touches the roof of the mouth, he recommends wearing a moist piece of cotton batting to fill up the space.

---

EVENING SESSION—SECOND DAY—AUGUST 1.

The first business was the election of officers, which resulted as follows :

President—S. H. Guilford, Philadelphia.

First Vice President—George Elliott, Meadville.

Second Vice President—James Truman, Philadelphia.

Recording Secretary—E. P. Kremer, Lebanon.

Assistant Secretary—Wm. B. Miller, Altoona.

Corresponding Secretary—W. H. Fundenberg, Pittsburgh.

Treasurer—G. W. Klump, Williamsport.

Board of Censors—Louis Jack, Wm. H. Trueman, W. E. Magill, C. S. Beck, J. Martin.

Drs. Magill and Gerhart were re-elected members of the State Examining Board.

Wilkesbarre was selected for the next meeting, commencing the last Tuesday in July, 1884.

The committee appointed to confer with the Examining Board presented, through their chairman, Dr. Jack, a report giving a brief history of the work of the Examining Board. Since it was established in 1876, two hundred and seventeen applicants for a certificate of qualification have appeared before them ; of this number twelve have been rejected. Some of these have afterwards attended a dental college and have graduated, and several have passed an examination the following year. The committee find that the examinations have been as rigid as those usually held at the colleges, and have no reason to think that any have received certificates who were not properly qualified. They desired at the same time to say most emphatically, that they do not consider the instruction of a preceptor equal to the systematic teaching of a college course in preparing a student for practice, and hope the time will soon come when all who desire to enter the profession will avail themselves of its advantages.

---

THIRD DAY—MORNING SESSION.

A discussion on Dr. Guilford's paper occupied some time, but nothing of special interest was brought out.

Dr. C. S. Beck moved that nothing in the shape of a clinic be brought before the society next year, unless it is entirely new. Adopted.

Dr. John A. Klump, of Delaware, read a paper on "The effect of Malaria Poisoning on the Dental Pulp." Practicing in a malarial district he had frequent opportunity of observing the effect of malarial poisoning upon the dental pulp. At first he was annoyed by patients coming to him suffering with severe tooth-ache, for which he could find no cause, and often met with cases where a number of sound teeth had been extracted in the hope of obtaining relief. This led him to closely study the matter, and by degrees he came to the conclusion that malarial poisoning had much to do with it. The next step was to try what effect the administration of anti-malarial remedies might have, and as these invariably gave relief, he felt safe in assigning malarial poisoning as the real cause of the trouble. In tracing its action he found malarial poison disturbed the circulation, and favored congestion and inflammation. When we consider the position of the pulp, enclosed in dense bony walls, we can readily see why it is so sensitive to any disturbance of its normal circulation or blood supply. As a result of these conditions, we first have inflammation and its accompanying odontalgia; this, if not relieved, is soon followed by strangulation at the apicial foramina, owing to congestion, and death of the pulp follows. He had no doubt this was the history of nearly all the dead pulps he found in sound teeth, and this also explained why, in teeth he had filled, he had found the pulps dead shortly after the patient had recovered from a malarial attack, and why he had so often failed in capping exposed pulps, although for a year or two they promised well. In these cases the previous injury, lowering the vitality of the teeth, made them more susceptible to injury, but why, when all the teeth were sound, one or two pulps should die and the rest escape, he did not know. Physicians practicing in his neighborhood have noticed that often the first symptom of malarial poison is dental irritation. This may occur sufficiently in advance, so that prompt administration of quinine will abort the attack. Whenever he meets with odontalgia of a neuralgic character for which he finds no visible cause, he prescribes three or four grains of quinine every three or four hours, until thirty grains have been taken, his object being to bring the patient under its influence as rapidly as possible. He finds it necessary to use large doses; probably smaller doses would suffice where malaria was not so prevalent. He also found advantage in using quinine in moderate doses for several days before dental operations, even when the patient is not suffering from malaria. Nerve capping he found very uncertain, especially between June and October. He had written the paper to call attention to the subject, with the desire to know if other dentists practicing in malarial districts have had the same experience.

DISCUSSION.

*Dr. C. S. Beck.*—Had met with several cases of odontalgia without any visible cause, which he had relieved with quinine, giving some twenty or thirty grains in the course of a day. The patients resided in a malarial district. Dr. Klump is entitled to credit for calling attention to the subject, and the care with which he has studied it.

*Dr. W. H. Trueman.*—Dr. Klump's paper is excellent and timely. The subject he has chosen is becoming more and more an important one to the dental and medical professions; not alone to those who practice in malarial districts, but to all. "Modern improvements" have introduced malaria into our city homes; it has invaded the parlor, the bed-chamber, the work-shop and office. Attentive readers of medical literature have noticed within the last few years, not only to what an alarming extent malaria in its various forms has become a formidable disease, but that it complicates and aggravates nearly all other diseases. No doubt many cases of tooth trouble for which we see no cause have been due to it, and no doubt some where we do recognize a cause would be relieved by moderate doses of quinine for a few days. He had no doubt in unhealthy districts "heroic" doses were needful, but in his practice he had found from three to six grains a day sufficient, and he often directed its use.

Several gentlemen spoke of having used quinine to relieve odontalgia with advantage, and were pleased that Dr. Klump had made such good use of his opportunities, as shown by the paper presented. Several questioned the propriety of such large doses, except in malarial districts, and thought harm would be done to give it so freely to patients not accustomed to its use, and where the malaria exists in a milder form.

*Dr. Magill.*—Called attention to the provision of the dental law requiring all dentists in the Commonwealth to register an affidavit in the recorder's office, stating how long they had been in practice, etc., and asked how that affidavit should be worded. After the matter had been talked over it was decided to request the committee on enforcement of dental law to have a proper form of affidavit prepared, and publish the same as soon as convenient. The form adopted is as follows:

STATE OF PENNSYLVANIA, }
County of } *ss.*

*Personally appeared before me* who being duly sworn according to law deposes and says that he now resides in the of county of ; that he has been in continuous practice of dentistry in this Commonwealth for the full term of years last past, as follows: In the of and county of from the month of 18 to the month of 18 , and in the of and county of from the month of 18 to the month of 18 ; and that said term of years is exclusive of the usual period of pupilage or study under instruction.

DEPONENT FURTHER STATES that he makes this statement for record in compliance with the Supplement to the Act of the General Assembly of Pennsylvania passed April 17, A. D. 1876, for the registration of dentists, etc.

*Sworn and subscribed before me*
*this day of* 18 }

The President elect, Dr. Guilford, after being installed in office, appointed the following committees:

Enforcement of Dental Law—W. E. Magill, J. W. Rhone, J. C. Green.

Dental Legislation—Drs. Litch, Miller, Jack, Robb, and Fundenberg.

Publication Committee—Drs. Magill, Kremer, Ansart, James Truman, Miller.

Executive Committee—C. S. Beck, E. D. Long, C. N. Pierce, G. L. Simpson, E. T. Darby.

The Society then adjourned to meet at Wilkesbarre, July 29, 1884.

